# Development of diabetes mellitus following hormone therapy in prostate cancer patients is associated with early progression to castration resistance

**DOI:** 10.1038/s41598-021-96584-1

**Published:** 2021-08-25

**Authors:** Tomonori Hayashi, Tomoyoshi Miyamoto, Noriaki Nagai, Atsufumi Kawabata

**Affiliations:** 1 Department of Pharmacy, Kindai University Nara Hospital, 1248-1 Otodacho, Ikoma, Nara 630-0293 Japan; 2 Laboratory of Pharmacology and Pathophysiology, Faculty of Pharmacy, Kindai University, 3-4-1 Kowakae, Higashi-Osaka, Osaka 577-8502 Japan; 3 Laboratory of Advanced Design for Pharmaceutics, Faculty of Pharmacy, Kindai University, 3-4-1 Kowakae, Higashi-Osaka, Osaka 577-8502 Japan

**Keywords:** Prostate cancer, Diabetes

## Abstract

To identify risk factors for the prognosis of prostate cancer (PC), we retrospectively analyzed the impact of lifestyle-related disorders as well as PC characteristics at initial diagnosis on the progression to castration-resistant PC (CRPC) in PC patients undergoing hormone therapy. Of 648 PC patients, 230 who underwent hormone therapy and met inclusion criteria were enrolled in this study. CRPC developed in 48 patients (20.9%). Univariate analysis using Cox proportional hazard model indicated that newly developed diabetes mellitus (DM) following hormone therapy (postDM), but not preexisting DM, as well as PC characteristics at initial diagnosis including prostate-specific antigen (PSA) ≥ 18 were significantly associated with the progression to CRPC. A similar tendency was also observed in the relationship between newly developed hypertension following hormone therapy and CRPC progression. On the other hand, neither dyslipidemia nor hyperuricemia, regardless the onset timing, exhibited any association with CRPC progression. In multivariate analysis, postDM and PSA ≥ 18 were extracted as independent risk factors for CRPC progression (adjusted hazard ratios, 3.38 and 2.34; *p* values, 0.016 and 0.019, respectively). Kaplan–Meier analysis and log-rank test clearly indicated earlier progression to CRPC in PC patients who developed postDM or had relatively advanced initial PC characteristics including PSA ≥ 18. Together, the development of lifestyle-related disorders, particularly DM, following hormone therapy, as well as advanced PC characteristics at initial diagnosis is considered to predict earlier progression to CRPC and poor prognosis in PC patients undergoing hormone therapy.

According to an epidemiologic study analyzing cancer incidence between 1985 and 2015 in three prefectures in Japan^1^, prostate cancer (PC) increased gradually before 2000, and quickly in early 2000s, which might be associated with the spread of prostate-specific antigen (PSA) screening, and thereafter remained as one of the most common cancers in Japanese males. Family history of PC is associated with the increased risk of PC morbidity by 2.4–5.6 times, suggesting the involvement of genetic factors^2^. Recent studies imply that 8–12% of PC cases involve a hereditary component associated with germline mutations or alterations in genes, such as BRCA1, BRCA2, HOXB13 or DNA MMR genes^3^. On the other hand, presumed acquired risk factors contributing to the development or poor prognosis of PC appear to include lifestyle-related diseases, such as hypertension (HT) and type 2 diabetes mellitus (DM) independent of obesity or serum triglycerides^4,5^, whereas plenty of conflicting evidence has been reported^6,7^.

Initial treatment options for PC after cancer staging and grading include prostatectomy, radiation therapy, and hormone therapy, especially androgen deprivation therapy (ADT)^8^. A majority of PC patients initially respond to traditional ADT, whereas most men with advanced PC eventually develop castration-resistant prostate cancer (CRPC). There is evidence that 10–20% of PC patients develop CRPC within a follow-up period of approximately 5 years^9^. Cancer cell proliferation in CRPC is considered to result from androgen receptor (AR) overexpression, AR mutations responsible for ligand-independent constitutive AR activity or promiscuous ligand activation of AR, intracrine steroid biosynthesis, acceleration of AR-independent mitogenic signals, etc.^10–12^. A recent clinical retrospective study demonstrated that the time to progression to CRPC following combined androgen blockade (CAB) therapy was shorter in high-risk PC survivors who had high PSA levels, high Gleason scores (GC), metastasis, etc. at initial diagnosis than non-high-risk PC men^13^. Hypogonadism in men may be associated with an increased cardiovascular risk^14^ and linked to metabolic syndromes including endothelial dysfunction, inflammation and abnormal glucose metabolism^15^. There is evidence that long-term ADT causes profound hypogonadism leading to constitutional symptoms, such as fatigue, vasomotor flushing, muscle mass loss, and osteo-sarcopenic obesity with fat mass gain accompanied by insulin resistance^16,17^. Preclinical studies have shown that high glucose levels may promote pathological consequences of cultured PC cells possibly through metabolic changes including altered protein glycosylation^18,19^. Of special interest is the retrospective clinical report that, in a subset of PC patients undergoing ADT without radiographic evidence of metastasis, those with DM progressed to CRPC more quickly than those without DM, although there was no such difference between DM and non-DM men in all PC patients with and without metastasis receiving ADT^5^. Nevertheless, there are no other clinical reports supporting this evidence. Collectively, the question, namely whether DM as well as initial PC stages/grades has impact on the time to CRPC development following hormone therapy, still remains to be answered or solved.

In the present study, to clarify the impact of preexisting or newly-developed metabolic disorders including DM, as well as the pathological characteristics of PC at the initial diagnosis, on the progression to CRPC, we conducted a retrospective cohort study in PC patients undergoing hormone therapy including ADT and CAB.

## Results

### Patient characteristics

Of the total 648 PC patients, 262 underwent hormone therapy including ADT and CAB, and 230 in consideration of the exclusion criteria were enrolled in this study. Patient characteristics are shown in Table 1. The median patient age was 76 years, and the median PSA was 17.6 ng/mL at initial diagnosis. Among 230 patients who underwent ADT, 190 (82.6%), 13 (5.7%) and 45 (19.6%) had administration of androgen receptor antagonists, surgical castration and radiotherapy, respectively. Lifestyle-related comorbidities including DM, hypertension (HT), dyslipidemia (DL) and hyperuricemia (HUA) in the enrolled PC patients were separated into preexisting DM, HT, DL and HUA before hormone therapy (preDM, preHT, preDL and preHUA, respectively) and post-therapy DM, HT, DL and HUA (postDM, postHT, postDL and postHUA, respectively) that newly developed following hormone therapy onset. There were 53 (23%) DM cases including 45 (19.6%) preDM and 8 (3.5%) postDM, 136 (59.1%) HT cases including 124 (53.9%) preHT and 12 (5.2%) postHT, 86 (37.4%) DL cases including 66 (28.7%) preDL and 20 (8.7%) postDL, and 53 (23%) HUA cases including 39 (17%) preHUA and 14 (6.1%) postHUA. Progression to CRPC occurred in 48 patients (20.9%) (Table 1). The duration [median months (range)] of ADT was 33.5 (2.23–226) in all patients, and there was no significant difference (*p*, 0.097) in the duration of ADT between CRPC and non-CRPC patients [37.8 (2.83–226) and 31.2 (2.23–148), respectively]. Metastasis-free survival could not be evaluated, because the present subjects included ones who had metastasis at the initial diagnosis.

**Table 1:** Characteristics of prostate cancer patients undergoing hormone therapy.

Characteristic	Classified group	
Patients who met inclusion criteria, *n*		230
Age (years), median (range)		76 (52–92)
Androgen receptor antagonist, *n* (%)	Yes No	190 (82.6) 40 (17.4)
Surgical castration, *n* (%)	Yes No	13 (5.7) 217 (94.3)
Radiotherapy, *n* (%)	Yes No	45 (19.6) 185 (80.4)
Stage at initial diagnosis, *n* (%)	I II III IV m.d	9 (3.9) 114 (49.6) 38 (16.5) 55 (23.9) 14 (6.1)
Gleason score at initial diagnosis, *n* (%)	≤ 6 7 ≥ 8 m.d	21 (9.1) 63 (27.4) 122 (53.0) 24 (10.4)
NCCN risk group at initial diagnosis, *n* (%)	Low Intermediate High	11 (4.8) 61 (26.5) 158 (68.7)
Metastasis at initial diagnosis, *n* (%)	Yes No	43 (18.7) 187 (81.3)
PSA (ng/mL) at initial diagnosis, median (range) *n* (%)		17.6 (3.86–12,275)
	≥ 18	112 (48.7)
	< 18	117 (50.9)
	m.d	1 (0.4)
Diabetes Mellitus (DM), *n* (%)	Yes preDM postDM No (nonDM)	53 (23.0) 45 (19.6) 8 (3.5) 177 (77.0)
Hypertension (HT), *n* (%)	Yes preHT postHT No (nonHT)	136 (59.1) 124 (53.9) 12 (5.2) 94 (40.9)
Dyslipidemia (DL), *n* (%)	Yes preDL postDL No (nonDL)	86 (37.4) 66 (28.7) 20 (8.7) 144 (62.6)
Hyperuricemia (HUA), *n* (%)	Yes preHUA postHUA No (nonHUA)	53 (23.0) 39 (17.0) 14 (6.1) 177 (77.0)
CRPC, *n* (%)	Yes No	48 (20.9) 182 (79.1)

*m.d*. missing data. Lifestyle-related diseases were separated into preexisting ones before hormone therapy (preDM, preHT, preDL and preHUA) and newly developed ones after the onset of hormone therapy (postDM, postHT, postDL and postHUA), and compared to cases without such diseases (nonDM, nonHT, nonDL and nonHUA).

### Relationship between CRPC progression and lifestyle-related diseases or PC characteristics at initial diagnosis

Data regarding the relationship between CRPC progression and clinical characteristics are shown in Table 2. Patients’ age, administration of androgen receptor antagonists, surgical castration and radiotherapy showed no significant association with CRPC progression. Then, the CRPC progression in PC patients who had preexisting and newly developed DM, HT, DL and HUA was compared with those who had no such diseases (nonDM, nonHT, nonDL and nonHUA, respectively). The univariate analysis showed significant association of CRPC progression with postDM [hazard ration (HR), 3.383; 95% confidence interval (CI), 1.31–8.76; *p*, 0.012], but not preDM. A similar tendency was observed in the relationship between CRPC progression and postHT, although not to the point of statistical significance (HR, 1.990; 95% CI, 0.88–4.49; *p*, 0.097). No such association was found for DL and HUA. Progression to CRPC was also significantly associated with PC characteristics at initial diagnosis, PSA ≥ 18 ng/mL (HR, 2.519; 95% CI, 1.26–5.03; *p*, 0.009), GS ≥ 8 (HR, 2.173; 95% CI, 1.05–4.51; *p*, 0.037), high risk in National Comprehensive Cancer Network (NCCN)’s classification (HR, 2.173; 95% CI, 1.10–5.52; *p*, 0.028), metastasis (HR, 4.256; 95% CI, 2.38–7.60; *p*, < 0.001), and Stage IV (HR, 7.089; 95% CI, 3.56–14.1; *p*, < 0.001). Multivariate analysis indicated an independent association of postDM (adjusted HR, 3.381; 95% CI, 1.26–9.11; *p*, 0.016) as well as PSA ≥ 18 ng/mL (adjusted HR, 2.342; 95% CI: 1.15–4.77; *p*, 0.019) with CRPC progression. In oncological characteristics, “high risk” was evaluated using PSA and GS, and the cancer stage and organ metastasis were strongly correlated with PSA. In addition, there are some statistical restriction of multivariate analysis because of the small size of samples. Therefore, we used propensity score adjustment to conduct multivariate analysis of the association of DM, HT, PSA, GS, high risk (NCCN), metastasis or stage IV with CRPC progression, and found a significant association of postDM with the time to CRPC, which was independent of other variables (HR, 3.95; 95%CI, 1.50–10.38; *p*, 0.0053) (Supplementary Table S1).

**Table 2:** Cox proportional univariate and multivariate analysis of the association between variables and CRPC progression.

Variables	Univariate analysis			Multivariate analysis		
	HR	95% CI or reference	*p* value	Adjusted HR	95% CI or reference	*p* value
Age ≥ 76 years	0.853	0.46–1.57	0.608			
Androgen receptor antagonist	0.611	0.30–1.47	0.176			
Surgical castration	1.367	0.57–3.29	0.486			
Radiotherapy	0.412	0.15–1.15	0.091			
nonDM	1.000	Reference	–	1.000	Reference	–
preDM	1.002	0.42–2.40	0.996	1.301	0.52–3.24	0.571
postDM	3.383	1.31–8.76	0.012*	3.381	1.26–9.11	0.016*
nonHT	1.000	Reference	–	1.000	Reference	–
preHT	0.861	0.45–1.63	0.646	1.010	0.51–1.99	0.977
postHT	1.990	0.88–4.49	0.097	2.055	0.89–4.76	0.093
nonDL	1.000	Reference	–			
preDL	0.610	0.28–1.33	0.212			
postDL	0.802	0.31–2.06	0.646			
nonHUA	1.000	Reference	–			
preHUA	1.455	0.67–3.16	0.343			
postHUA	1.326	0.47–3.77	0.596			
PSA ≥ 18 ng/mL	2.519	1.26–5.03	0.009*	2.342	1.15–4.77	0.019*
GS ≥ 8	2.173	1.05–4.51	0.037*			
High risk in NCCN’s	2.461	1.10–5.52	0.029*			
Metastasis	4.256	2.38–7.60	< 0.001*			
Stage IV	7.089	3.56–14.1	< 0.001*			

*PSA* prostate-specific antigen, *GS* Gleason score, *NCCN’s* The National Comprehensive Cancer Network’s classification, *HR* hazard ratio, *CI* confidence interval. *Statistically significant (*p* < 0.05).

### Association between the time to CRPC progression and lifestyle-related diseases or PC characteristics at initial diagnosis

**Figure 1:**
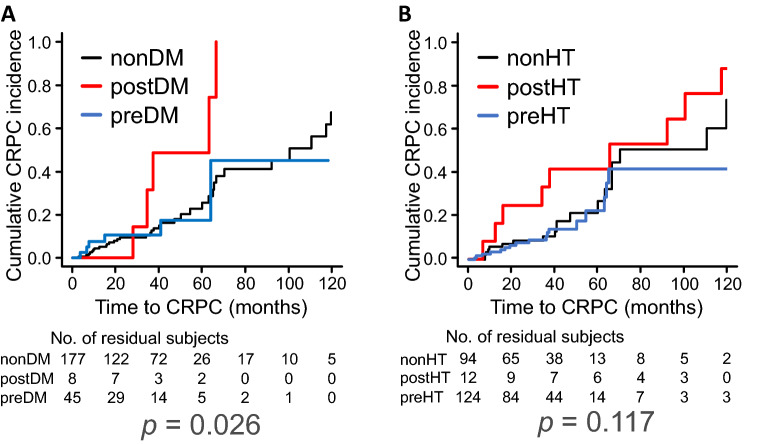
Impact of DM and HT on the time to CRPC progression in PC patients undergoing hormone therapy. DM and HT were separated into preexisting ones (preDM and preHT) and newly developed ones (postDM and postHT). Kaplan–Meier curves show the time-related progression to CRPC in patients who had preDM, postDM and no DM (nonDM) (**A**) and who had preHT, postHT and no HT (nonHT) (**B**). Statistical differences among groups were analyzed by the log-rank test. *p* values are shown in the bottom of each graph.

Kaplan–Meier curves for the cumulative incidence of CRPC progression in nonDM, preDM and postDM groups clearly showed the earlier progression to CRPC in PC patients who had postDM, and the log-rank test indicated significant differences among those groups (log-rank *p*,0.026) (Fig. 1A). The median time (months) to CRPC onset in nonDM and postDM groups was 100.4 (95% CI, 66.4–124.5) and 63.2 (95% CI, 28.1-NA), respectively. No such clear or significant association of the time to CRPC with HT was detected in Kaplan–Meier curves or log-rank test (Fig. 1B). There was also no significant association of DL and HUA with the time to CRPC (Supplementary Fig. S1).

**Figure 2:**
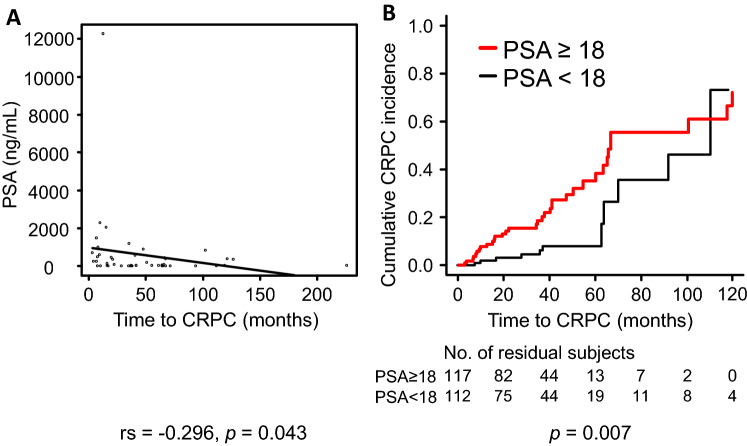
Impact of PSA levels at initial diagnosis on the time to CRPC progression in PC patients undergoing hormone therapy. (**A**) Correlation between PSA levels and the time to CRPC progression. Spearman’s rank correlation coefficient (rs) and *p* values are shown in the bottom of the graph. Box-and-whisker diagrams for PSA and the time to CRPC progression are shown outside the graph. (**B**) Kaplan–Meier curves show the time-related progression to CRPC in patients with PSA ≥ 18 ng/mL or PSA < 18 ng/mL at initial diagnosis. Statistical differences among groups were analyzed by the log-rank test. *p* values are shown in the bottom of each graph.

A significant negative correlation was observed between initial PSA levels and the time to CRPC progression (rs, − 0.296; *p*, 0.043) (Fig. 2A). The Kaplan–Meier curve and log-rank test indicated that initial PSA levels ≥ 18 ng/mL were significantly associated with earlier progression to CRPC (log-rank *p*, 0.007) (Fig. 2B). The median time (months) to CRPC was 66.4 (95% CI, 60.0–119.6) and 110.4 (95% CI, 63.9-NA) in patients with PSA of ≥ 18 ng/mL and of < 18 ng/mL, respectively. Similarly, the time to CRPC was significantly associated with other PC characteristics at initial diagnosis, such as GS ≥ 8 (log-rank *p*, 0.033), high risk group in NCCN’s classification (log-rank *p*, 0.024), distant metastasis (log-rank *p*, < 0.001), and Stage IV (log-rank *p*, < 0.001) (Supplementary Fig. S2).

## Discussion

The present study, for the first time to our knowledge, demonstrates the significant association of postDM, but not preDM, with the progression to CRPC, and also ascertains the impact of initial diagnostic characteristics of PC, including high PSA levels, GS, high risk in NCCN’s classification and stages, in addition to metastasis, on CRPC progression, in agreement with the recent study^13^. Our study also shows that postDM is a risk factor for CRPC progression, which is independent of HT and initial pathological characteristics of PC including PSA, GS, NCCN’s classification, stages and metastasis.

Previous clinical studies on the relationship between lifestyle-related metabolic disorders, particularly DM, and PC appear controversial^4–7^. A retrospective clinical report has shown the association of CRPC progression with DM in a subset of PC patients who had no radiographic evidence of metastasis, but not in all PC patients. This is understandable, considering the present results indicating that metastasis itself is significantly correlated with CRPC progression (see Table 2 and Supplementary Fig. S2C), in agreement with an independent report^13^. The significant association between progression to CRPC and postDM, but not preDM, in the present study (see Table 2 and Fig. 1A) is consistent with a recent study reporting that elevated fasting blood glucose levels after PC diagnosis, but not those before PC diagnosis, were associated with an increased risk of PC death^20^.

Previous studies have suggested that long-term ADT may increase the risk of developing insulin resistance and hyperglycemia in PC patients^21–23^. Animal studies have shown that testosterone increased glucose-stimulated insulin secretion (GSIS) via activation of AR expressed in β cells, and the activated AR amplified the insulin secretagogue effect of glucagon-like peptide 1, and that male mice lacking AR had lower GSIS, thereby suggesting that the decreased AR signaling following ADT is associated with the pathophysiology of DM^24^.

It is noteworthy that, in the present study, PC patients who developed HT after the onset of hormone therapy also tended to have a higher risk of CRPC progression (see Table 2 and Fig. 1). There is also evidence that a decrease in testosterone may contribute to the development of HT and that ADT may increase the prevalence of HT in PC patients, especially among those using gonadotropin-releasing hormone (GnRH) receptor agonists^25,26^. In this context, the development of lifestyle-related diseases including DM and HT following ADT or CAB could be a risk factor for earlier progression to CRPC.

Fibroblast growth factor (FGF) family, such as FGF19, FGF21, and FGF23, are considered metabolic regulators^27–29^. Circulating FGF21 levels increase with insulin resistance^30^, and dysfunction of FGF21 receptor signaling may be associated with lifestyle-related diseases such as HT and type 2 DM^31^. FGF family also plays a role in the progression of PC^32^. FGF8 and FGF23 promote the growth and progression of PC^33,34^. There is also evidence that FGF bypasses a functional requirement for AR after AR antagonism, and that pharmacological blockade of FGF receptors suppresses CRPC growth^35^. It is thus hypothesized that the induction of FGF following the development of lifestyle-related diseases including DM in PC patients undergoing hormone therapy might contribute to the development of CRPC from PC.

The limitation of this study is related to its retrospective nature, the small sample size of patients, and the lack of detailed data regarding obesity and metabolic syndrome. The retrospective nature limits the ability to control confounding factors, although we used propensity score analysis to consider confounding factors in the present study. Prospective and/or multicenter studies in future are needed to ascertain the present evidence.

In conclusion, to the best of our knowledge, this study provides the first clinical evidence that the development of lifestyle-related diseases, especially DM, is associated with early progression to CRPC, and ascertains the impact of initial diagnostic characteristics of PC on CRPC progression, as described recently^13^.

## Methods

### Patient selection, data collection and CRPC definition

Among patients who were diagnosed with PC at Kindai University Nara Hospital from April 2014 to August 2018, those who underwent hormone therapy including ADT and CAB, regardless of receiving operation, were selected, and their information from medical records were collected and analyzed. Exclusion criteria were: (1) the period of hormone therapy was shorter than 2 months; (2) insufficient or unusual hormone therapy, such as hormone therapy only before surgery or including a hormone therapy suspension period for 6 months or more. ADT in the present patients included administration of leuprorelin and goserelin, gonadotropin-releasing hormone (GnRH) receptor agonists that cause GnRH receptor desensitization, and degarelix, a GnRH receptor antagonist. Patients who underwent CAB received androgen receptor antagonists, such as bicalutamide, flutamide and chlormadinone, in addition to ADT. CRPC was defined essentially according to the criteria of the European Urology Guidelines^36^, as follows: in PC patients with serum testosterone levels below 50 ng/dL, those who had 3 consecutive rises of PSA levels measured at intervals of 1 week or more, resulting in two 50% rises from the PSA nadir together with PSA levels higher than 2.0 ng/mL, or who had the exacerbation of existing lesions or appearance of new lesions on radiological images.

### Study design, statistical analyses of clinical data and ethical approval

Selected PC patients undergoing hormone therapy were classified at the initial diagnosis by cancer stages (I–III vs. IV), GS (< 8 vs. ≥ 8), metastases, PSA levels (categorized by median splits), and the National Comprehensive Cancer Network (NCCN) risk classification (‘low’ or ‘medium’ risk vs. ‘high’ risk)^37^. The presence of lifestyle-related comorbidities including DM, hypertension (HT), dyslipidemia (DL) and hyperuricemia (HUA) in the PC patients was examined by checking their diagnostic records and prescribed drugs. Considering the timing of the disease onset, they were separated into preexisting diseases before hormone therapy (preDM, preHT, preDL and preHUA) and newly developed diseases after the onset of hormone therapy (postDM, postHT, postDL and postHUA), and compared with PC patients who had no such disorders (nonDM, nonHT, nonDL and nonHUA). Difference of the duration of ADT in CRPC and non-CRPC patients was statistically analyzed by Mann–Whitney’s U test. Statistical comparisons of CRPC progression between categorized groups were performed by univariate and multivariate analyses using Cox proportional hazard model, and respective HR and adjusted HR are shown with 95% CI. Time to CRPC progression in different groups was analyzed using the Kaplan–Meier curve and tested by the log-rank test. Spearman's rank correlation coefficient was used to examine the correlation between PSA levels and the time to CRPC progression. Further, considering statistical restriction of multivariate analysis because of the small size of samples, we used propensity score adjustment in order to evaluate statistical significance of the independent association of DM, HT, PSA, GS, high risk (NCCN), metastasis or stage IV with the time to CRPC progression. Briefly, the propensity score was used as a covariate in multivariate analysis of the independent effect of each factor, to keep statistical power. All reported *p* values were bilateral, and *p* < 0.05 was considered statistically significant. All statistical analyses were performed using EZR (Saitama Medical Center, Jichi Medical University, Japan)^38^, a pilot user interface for R (version 1.41).

The study protocol was in accordance with the relevant guidelines and regulations including the Declaration of Helsinki, and approved by the Ethics Committee of Kindai University Nara Hospital (approval number 538). Owing to the retrospective nature of this study, the need for informed consent was waived by the Ethics Committee of Kindai University Nara Hospital, which approved the use of an opt-out strategy with respect to patient consent; i.e. patients were included in the research unless they expressly requested to be excluded. The hospital’s official website was used for communication of the information about our research with the patients. In addition, we confirm that any member of our research team named in the author list did not have access to identifying patient information when analyzing the data.

## Supplementary Information

Supplementary Information 1.

Supplementary Information 2.

## Abbreviations

ADT: Androgen deprivation therapy
AR: Androgen receptor
CAB: Combined androgen blockade
CI: Confidence interval
CRPC: Castration-resistant prostate cancer
DL: Dyslipidemia
DM: Diabetes mellitus
FGF: Fibroblast growth factor
GnRH: Gonadotropin-releasing hormone
GS: Gleason score
HR: Hazard ratio
HT: Hypertension
HUA: Hyperuricemia
NCCN: The National Comprehensive Cancer Network
PC: Prostate cancer
preDM: Preexisting diabetes mellitus
preHT: Preexisting hypertension
preDL: Preexisting dyslipidemia
preHUA: Preexisting hyperuricemia
postDM: Post-therapy diabetes mellitus
postHT: Post-therapy hypertension
postDL: Post-therapy dyslipidemia
postHUA: Post-therapy hyperuricemia
PSA: Prostate-specific antigen
