# Supplementary material for: Development of diabetes mellitus following hormone therapy in prostate cancer patients is associated with early progression to castration resistance
Source: Sci Rep. 2021 Aug 25;11:17157. doi: 10.1038/s41598-021-96584-1 (PMC8387479; doi:10.1038/s41598-021-96584-1)
Supplement: Supplementary file 1 — Supplementary Information 1. [file 41598_2021_96584_MOESM1_ESM.pdf]

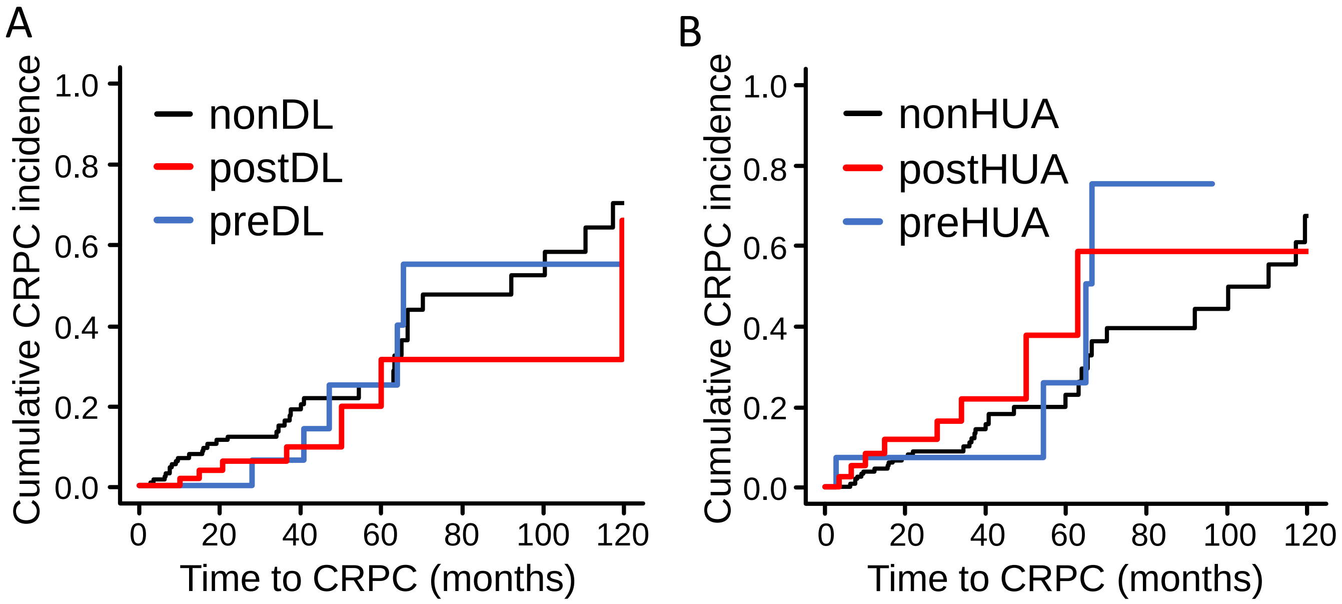

No. of residual subjects

nonDL	144	96	56	22	12	8	4
postDL	20	16	13	5	3	1	0
preDL	66	46	20	6	4	2	1

p = 0.434

No. of residual subjects

nonHUA	177	126	70	25	16	10	4
postHUA	14	11	8	4	1	0	0
preHUA	39	21	11	4	2	1	1

p = 0.584

Supplementary Figure S1. The time to CRPC progression in PC patients undergoing hormone therapy with or without DL or HUA. DL and HUA were separated into preexisting ones (preDL and preHUA) and newly developed ones (postDL and postHUA). Kaplan-Meier curves show the time-related progression to CRPC in patients who had preDL, postDL and no DL (nonDL) (A) and who had preHUA, postHUA and no HUA (nonHUA) (B). Statistical differences among groups were analyzed by the log-rank test. *p* values are shown in the bottom of each graph.

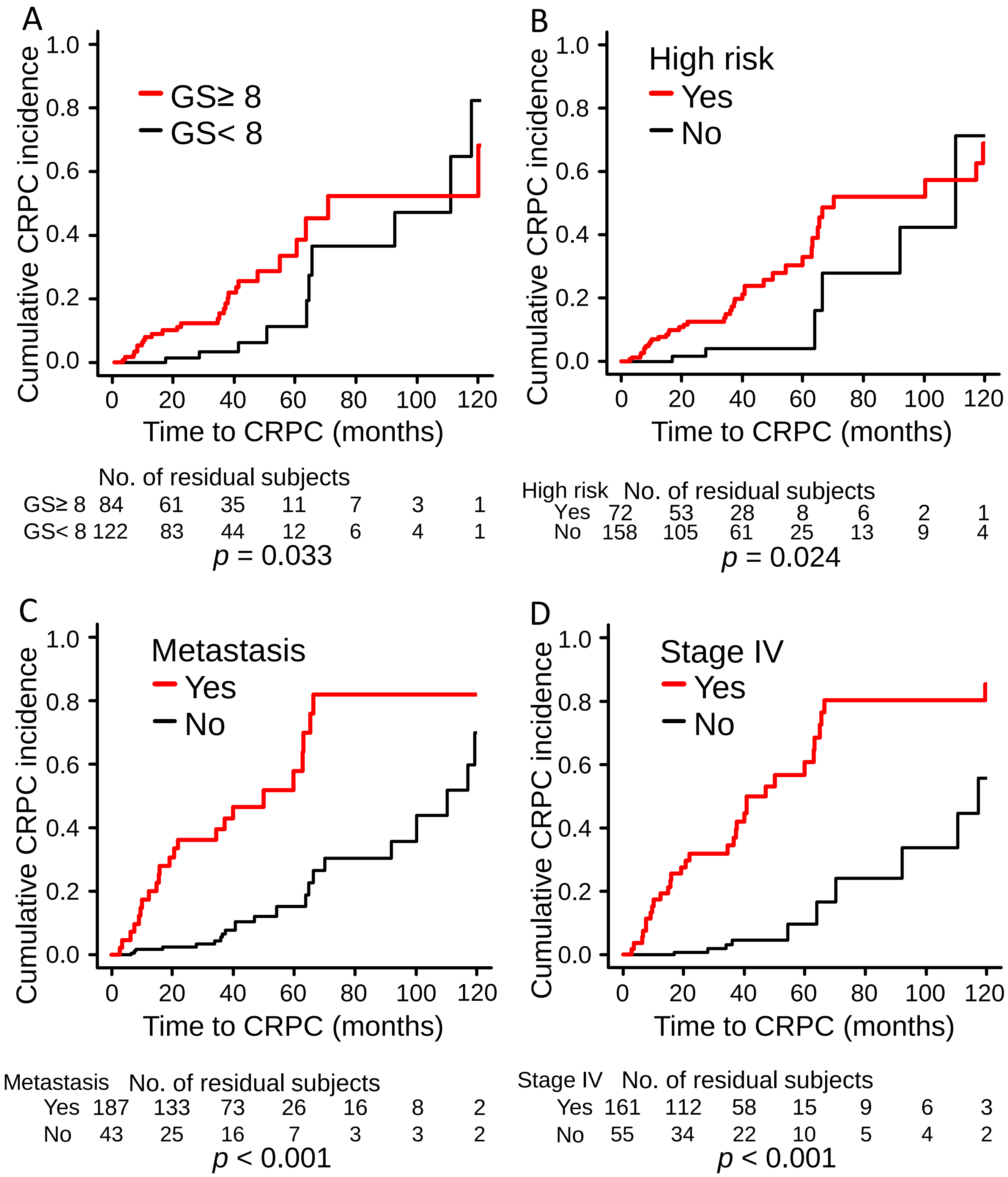

Supplementary Figure S2. Impact of PC characteristic at initial diagnosis on the time to CRPC progression in PC patients undergoing hormone therapy. Kaplan-Meier curves show the time-related progression to CRPC in patients who had Gleason score (GS) ≥ 8 or GS < 8 (A), high risk or not in NCCN classification (B), metastasis or not (C) and stage IV or not (I-III) (D) at initial diagnosis. Statistical differences between groups were analyzed by the log-rank test. *p* values are shown in the bottom of each graph.
