# Supplementary material for: Development of diabetes mellitus following hormone therapy in prostate cancer patients is associated with early progression to castration resistance
Source: Sci Rep. 2021 Aug 25;11:17157. doi: 10.1038/s41598-021-96584-1 (PMC8387479; doi:10.1038/s41598-021-96584-1)
Supplement: Supplementary file 2 — Supplementary Information 2. [file 41598_2021_96584_MOESM2_ESM.pdf]

Variables	Multivariate analysis		
	Adjusted HR	95%CI or reference	*p* value
nonDM	1.00	Reference	-
preDM	1.83	0.73 - 4.61	0.198
postDM	3.95	1.50 - 10.38	0.005*
nonHT	1.00	Reference	-
preHT	1.19	0.61 - 2.29	0.612
postHT	2.18	0.98 - 4.88	0.057
PSA ≥ 18 ng/mL	1.08	0.49 - 2.37	0.847
GS ≥ 8	1.46	0.69 - 3.07	0.326
High risk in NCCN's	1.46	0.59 - 3.59	0.413
Metastasis	1.27	0.57 - 2.82	0.551
Stage IV	5.73	2.45 - 13.41	< 0.001*

Supplementary Table S1. The independent association of DM, HT, PSA, GS, high risk (in NCCN's classification), metastasis or Stage IV with CRPC progression using the propensity score. Cox proportional multivariate analysis of the association of each factor with CRPC progression was conducted after propensity score adjustment, in which the propensity score was separately calculated as a function of the other candidate risk factors and used as an adjustment covariate. *Statistically significant ($p < 0.05$).
